# Bibliometric Analysis of Research on Lip Prints

**DOI:** 10.7759/cureus.75950

**Published:** 2024-12-18

**Authors:** Rahul Krishna RV, Sujitha Ponraj, Kavitha Ramar, Victor Samuel A, Rajakumar S, Anitha Annadurai, Arya Acca Varghese

**Affiliations:** 1 Department of Pediatric and Preventive Dentistry, SRM Kattankulathur Dental College and Hospital, SRM Institute of Science and Technology, Chennai, IND

**Keywords:** bibliometric analysis, cheiloscopy, forensic identification, forensic odontology, lip print analysis

## Abstract

Lip prints, or cheiloscopy, are unique patterns of grooves and wrinkles, gaining prominence in forensic science as reliable tools for personal identification, akin to fingerprints and DNA profiling. Advances in imaging techniques have enhanced their forensic applicability. This study conducts a bibliometric analysis to explore global research trends, key contributors, and thematic developments in lip print research. The search was done with a range of databases like Web of Science, Scopus, and PubMed in September 2024. Included were pertinent studies on lip print analysis, and studies that were irrelevant were excluded. Specific study characteristics, citations, and years of research publication were studied using VOSviewer (Centre for Science and Technology Studies, Leiden University, The Netherlands) and Biblioshiny (RStudio (Posit PBC, Boston, MA, US)) to examine various networks and themes of identity. Among the 5,864 articles retrieved, 198 studies were chosen for a detailed bibliometric analysis. The volume of research output has been on the rise since 1971. India leads with 133 publications backed by Saveetha University and Ajman University. Terms like “forensic identification” or “cheiloscopy” show an interest in the area of humans as an object of study. Collaborative networks are still unevenly developed and only begin to have international partnerships. This leading role of India is indicative of the importance Indian researchers are placing on forensic odontology as evidenced by the existence of regional journals and strong institutions. Foundational studies are still relevant today, though their frequency of citation per article has been decreasing as the discipline progresses. This bibliometric analysis presents the changes that have taken place over time in the field of lip print study and a particular focus on the input and collaboration of different regions of the world. Although some limitations have been noted, these results have implications in the development of forensic odontology as well as in the direction of equitable research development across the globe.

## Introduction and background

Lip prints refer to the patterns of lines and wrinkles on human lips. This gave a considerable interest in studying lip prints as part of forensic science and personal identification. From the landmark work of Dr. Edmond Locard in the early 1900s, cheiloscopy or the study of lip prints has received much attention mainly because it could be useful in criminal investigations and biometric identification. Due to the uniqueness and permanence thereof, lip prints are a conclusive means of identifying individuals like fingerprints and DNA profiles [1].

Bibliometric analysis is a great tool that helps assess how, when, and in what areas growth has occurred as well as the significance and emerging trends of any research domain. The full paper reviews the literature on lip prints and provides valuable insights into the history of this specialized area of forensic science, current research hotspots within it, and possible directions for future exploration [2]. The literature sheds light on the importance of lip prints in various contexts such as criminal investigations, disaster victim identification, and genetic research [3].

A lot of research has been dedicated to establishing the uniqueness and classification of lip print patterns. Suzuki and Tsuchihashi [1] were the pioneers to propose a classification system that is based on groove shapes and arrangement. Subsequent studies have reaffirmed the uniqueness of lip prints as well as their effectiveness in forensic identification [4,5]. Imaging technology advancements and software have made accuracy and reliability in lip print analysis in accordance with integration into forensic procedures more effective [6]. Studies mentioned have also contributed to understanding how factors such as age, gender, and environmental conditions influence variability and stability at different times of lip print patterns [7,8]. All these works are an interdisciplinary study of lip prints; from information technology, anthropology, and genetics to forensic science.

This study attempts to provide an exhaustive bibliometric analysis of lip print research relating to the trends in publication, principal authors and institutions, and changes in thematic focus. We clarify the evolution of lip print research through a detailed study of scientific literature and point out contributions that have shaped this field. This work will provide a historical context regarding the subject and assist in pinpointing areas where there are gaps as well as opportunities for further research on cheiloscopy.

## Review

Materials and methods

A comprehensive search was conducted in the Web of Science, Scopus database, and PubMed on September 30, 2024, with no language restrictions. The search keywords included “lip prints”, “cheiloscopy”, “forensic identification”, “forensic odontology”, and “biometric identification”. For inclusion, all types of studies published in the English language related to lip print analysis and its forensic applications were considered, regardless of publication status or date. Exclusion criteria were applied to studies that focused on other forms of identification unrelated to lip prints or those lacking sufficient relevance to the scope of the analysis. Two reviewers (SP and RV) independently reviewed the titles and abstracts of all identified publications. Full texts were examined when abstracts did not provide sufficient information. In cases of disagreement between the two reviewers, a third reviewer (KR) was consulted. The status of the publication or its date was not restricted. The extracted information, reviewed independently by two reviewers (SP and RV), included characteristics of the study and its citation information like the name of the journal, title of the article, year of its publication, and citations), as well as the study design and topics addressed. Analysis and network visualization of the authors, countries, and keywords were performed using VOSviewer software (version 1.6.13) (Centre for Science and Technology Studies, Leiden University, The Netherlands) and Biblioshiny (version 4.0) (RStudio (Posit PBC, Boston, MA, US)).

Results

Main Information

**Figure 1:**
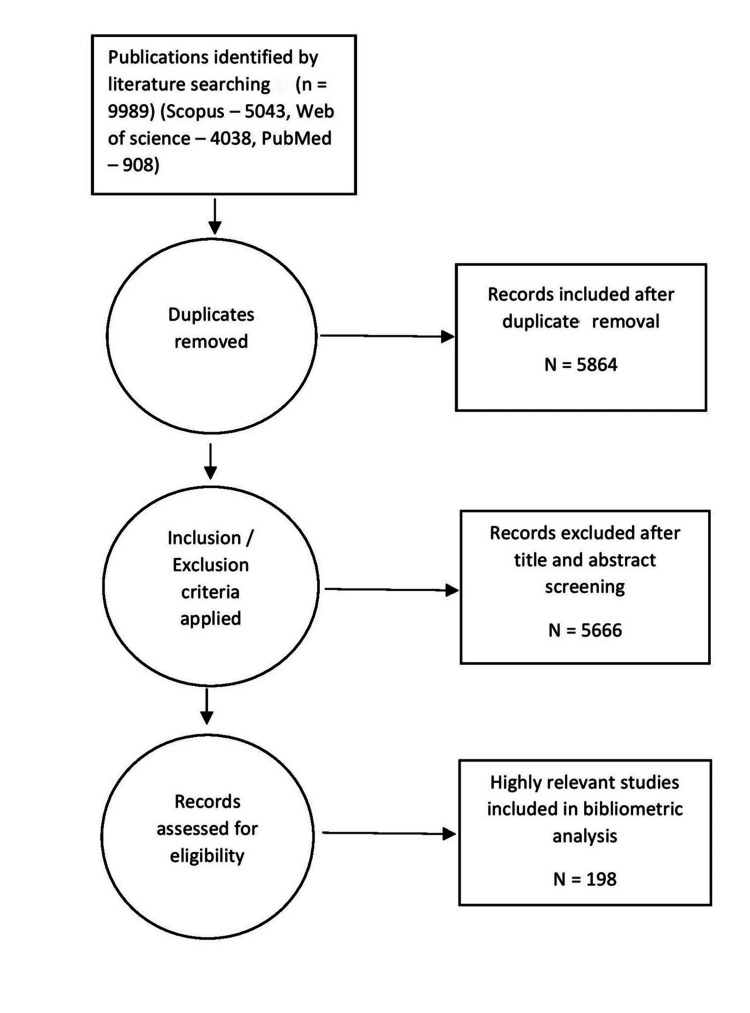
Flowchart on the inclusion of articles

Figure 1 and Table 1 present the descriptive data of scientific evidence on the subject. A total of 5,864 articles were included after duplicates were removed, with 198 highly relevant studies selected for bibliometric analysis. The earliest publication in this field dates back to 1971, with a steady increase in research activity thereafter. The publications showed an annual growth rate of 3.08%. In total, 684 authors contributed to these publications, with an average of 3.81 co-authors per document. Among the 198 highly relevant studies, the majority were original articles, with a smaller proportion of review articles.

**Table 1:** Main information

Timespan	1971:2024
Sources (journals, books, etc.)	102
Documents	198
Annual growth rate %	3.08
Document average age (year)	7.62
Average citations per doc	12.17
References	6,451
Document contents	
Keywords plus (ID)	702
Author's keywords (DE)	363
Authors	
Authors	684
Authors of single-authored docs	11
Author collaboration	
Single-authored docs	12
Co-authors per doc	3.81
International co-authorships %	9.596
Document types	
Article	150
Book	5
Book chapter	4
Conference paper	5
Letter	3
Note	1
Review	29
Short survey	1

Overall Growth Trends

**Figure 2:**
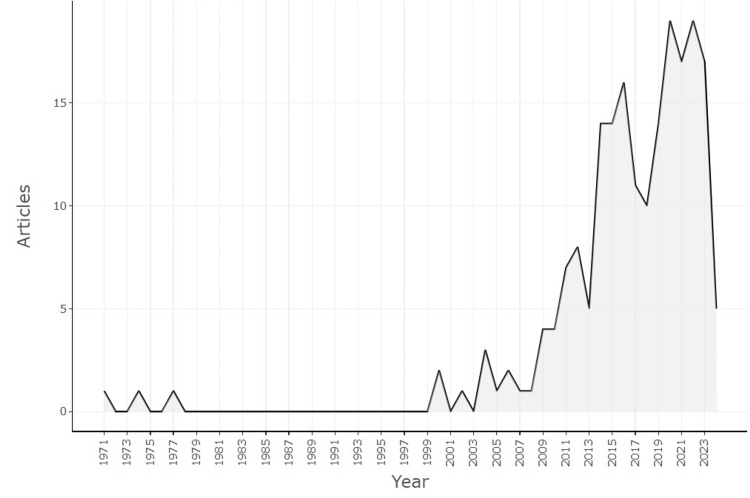
Annual scientific production

Figure 2 shows the progression of this field over time. The earliest work, published in 1971, indicated slight development during the preceding years. Between 1971 and 2003, only six works were published, signifying a relatively slow growth rate during this period. In stark contrast to the previous period, an explosive growth rate in publications was observed after the year 2004. It was also noted for the years 2020 (total publication (TP) = 19) and 2022 (TP = 19), indicating sustained growth in publication and citation as well. This is scientific output data showing that researchers are increasingly interested in and advancing knowledge in this area.

Citations

Table 2 captures the trend of lip print research from 1971 to 2024. In the early years, such as in 1971 and 1974, there were occasional but well-cited articles, resulting in average citations of 68 and 190 per article, respectively. A significant increase in research activity was noted after the year 2000, which continued to peak with the publication of 19 articles each in the years 2020 and 2022. However, while it has grown during recent years, the average citations per article have declined during those years, indicative of a shift toward more publications with less impactful citations. It underscores both increasing prominence and changes in research orientation within the field.

**Table 2:** Annual publishing and citation details TC: total citation

Year	Mean TC per Art	Mean TC per year	Citable years
1971	1	68.00	1.28
1972	0	0.00	0.00
1973	0	0.00	0.00
1974	1	190.00	3.80
1975	0	0.00	0.00
1976	0	0.00	0.00
1977	1	11.00	0.23
1978	0	0.00	0.00
1979	0	0.00	0.00
1980	0	0.00	0.00
1981	0	0.00	0.00
1982	0	0.00	0.00
1983	0	0.00	0.00
1984	0	0.00	0.00
1985	0	0.00	0.00
1986	0	0.00	0.00
1987	0	0.00	0.00
1988	0	0.00	0.00
1989	0	0.00	0.00
1990	0	0.00	0.00
1991	0	0.00	0.00
1992	0	0.00	0.00
1993	0	0.00	0.00
1994	0	0.00	0.00
1995	0	0.00	0.00
1996	0	0.00	0.00
1997	0	0.00	0.00
1998	0	0.00	0.00
1999	0	0.00	0.00
2000	2	58.50	2.44
2001	0	0.00	0.00
2002	1	12.00	0.55
2003	0	0.00	0.00
2004	3	128.00	6.40
2005	1	35.00	1.84
2006	2	20.00	1.11
2007	1	189.00	11.12
2008	1	59.00	3.69
2009	4	22.00	1.47
2010	4	14.00	1.00
2011	7	10.57	0.81
2012	8	12.00	1.00
2013	5	18.20	1.65
2014	14	8.07	0.81
2015	14	14.86	1.65
2016	16	7.06	0.88
2017	11	11.36	1.62
2018	10	4.80	0.80
2019	14	4.71	0.94
2020	19	2.00	0.50
2021	17	5.53	1.84
2022	19	2.47	1.24
2023	17	2.41	2.41
2024	5	1.20	-

Leading Countries

Table 3 highlights the contributions of various countries to dental age estimation research. India leads with 133 articles and 715 total citations, exhibiting a notable multiple-country publication (MCP) ratio of 0.037. Saudi Arabia and Chile show strong collaboration with MCP ratios of 0.333 and 0.667, respectively. Countries like Spain and Portugal, despite fewer articles, achieve significant citation impacts with 189 and 220 total citations. Other nations, such as Brazil and Indonesia, contribute exclusively through single-country publications (SCPs). The dataset underscores a mix of high-output countries and smaller contributors with varying levels of collaboration and impact.

**Table 3:** Country publication details TC: total citation; SCP: single-country publication; MCP: multiple-country publication; Freq: frequency

Country	Articles	TC	SCP	MCP	Freq	MCP ratio
India	133	715	79	3	0.414	0.037
Saudi Arabia	6	99	4	2	0.03	0.333
Spain	6	189	6	0	0.03	0
Malaysia	5	31	3	2	0.025	0.4
Brazil	4	22	4	0	0.02	0
Indonesia	4	15	4	0	0.02	0
Argentina	3	4	2	1	0.015	0.333
Chile	3	29	1	2	0.015	0.667
Nepal	3	7	3	0	0.015	0
Poland	3	35	3	0	0.015	0
Portugal	3	220	3	0	0.015	0
USA	3	7	3	0	0.015	0
Egypt	2	33	2	0	0.01	0
Iraq	2	2	1	1	0.01	0.5
Italy	2	15	2	0	0.01	0
Nigeria	2	11	2	0	0.01	0
Australia	1	54	1	0	0.005	0
Colombia	1	4	1	0	0.005	0
Croatia	1	5	1	0	0.005	0
Cyprus	1	0	1	0	0.005	0
France	1	12	1	0	0.005	0
Ghana	1	5	0	1	0.005	1
Iran	1	15	1	0	0.005	0
Japan	1	16	1	0	0.005	0
Lithuania	1	2	1	0	0.005	0
Macedonia	1	0	0	1	0.005	1
Pakistan	1	0	1	0	0.005	0
South Africa	1	8	1	0	0.005	0
Thailand	1	0	1	0	0.005	0
Turkey	1	3	1	0	0.005	0

Leading Institutions

**Figure 3:**
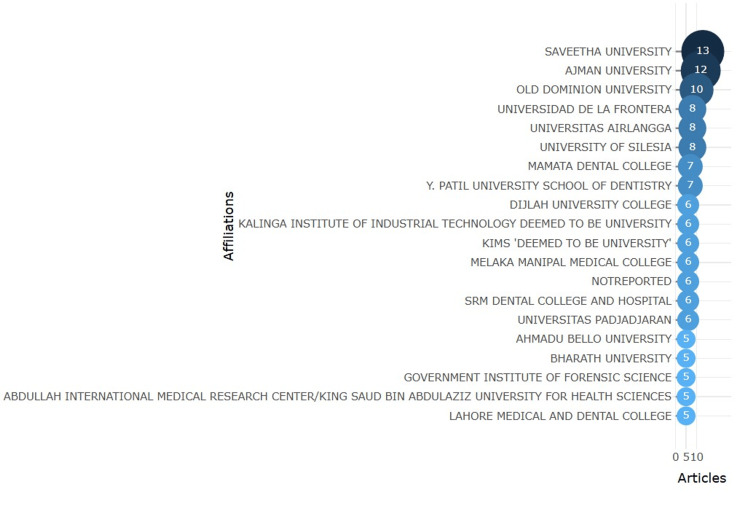
Institutions with the highest number of publications

Figure 3 highlights the top 20 most active affiliations in lip print research. Saveetha University leads with 13 publications, followed by Ajman University with 12, and Old Dominion University with 10. Other prominent contributors include Universidad de La Frontera and Universitas Airlangga, each with eight publications. Institutions like Y. Patil University School of Dentistry, Mamata Dental College, and the Kalinga Institute of Industrial Technology also demonstrate strong research activity, with six to seven publications each. This data underscores the global and institutional diversity driving advancements in the field.

Authors

Tables 4, 5 highlight significant contributions to lip print research, showcasing key metrics like article counts, fractionalized contributions, h-index, g-index, m-index, total citations, and publication history. Gupta S leads with five articles and a fractionalized contribution of 1.33, while Castelló A, Fonseca GM, and Verdú F each have four articles with similar fractionalized contributions and impactful records, including an h-index and g-index of four, with 85 citations since 2002. Notable contributors like Chidambaram R (2.00) and Acharya AB (1.50) reflect significant engagement, while Caldas IM stands out with the highest total citations at 220, despite modest h-index and g-index values of two and three. Recent contributors, such as Daruka Prasad B, Malleshappa J, and Mamatha GR, show promising activity in 2023 with an m-index of one. Emerging researchers like Abirami MS and Adamu LH further illustrate a collaborative and evolving research landscape, with diverse and impactful contributions advancing the field.

**Table 4:** Top 50 authors with the highest number of publications

Authors	Articles	Articles fractionalized
Gupta S	5	1.33
Castelló A	4	1.33
Fonseca GM	4	1.50
Verdú F	4	1.33
Badiye A	3	1.20
Caldas IM	3	1.08
Kadashetti V	3	0.81
Kapoor N	3	1.20
Acharya AB	2	1.50
Al-Gaidi SA	2	0.30
Almutairi AF	2	0.50
Alvarez-Seguí M	2	0.67
Alvarez M	2	0.67
Belgaumi U	2	0.31
Bhuyan L	2	0.37
Bommanavar S	2	0.31
Chatterjee P	2	0.67
Chidambaram R	2	2.00
Crispino F	2	0.67
Daruka Prasad B	2	0.23
Doroz R	2	0.45
Fernandes LCC	2	0.50
Fernandes R	2	0.75
Gupta OP	2	0.58
Houck M	2	0.67
Jagannath GV	2	0.50
Jha K	2	0.50
Kasprzak J	2	0.75
Kumar S	2	0.67
Kurniawan A	2	0.39
Malleshappa J	2	0.23
Mamatha GR	2	0.23
Mcadam T	2	0.67
Melvin W	2	0.40
Nagabhushana H	2	0.23
Nagrale N	2	0.45
Ninave S	2	0.50
Oliveira JA	2	0.50
Patond S	2	0.45
Pollo-Cattaneo MF	2	0.67
Porwik P	2	0.45
Rabello PM	2	0.50
Radha Krushna BR	2	0.23
Saha S	2	0.50
Sahana S	2	0.50
Salam M	2	0.50
Samadi F	2	0.40
Sandhya S	2	0.75
Shamim T	2	1.25
Sharma P	2	0.58

**Table 5:** Top 50 authors with the highest citations TC: total citation; NP: number of publications; PY: publication year

Authors	h-index	g-index	m-index	TC	NP	PY start
Castelló A	4	4	0.174	85	4	2002
Verdú F	4	4	0.174	85	4	2002
Al-Gaidi SA	2	2	0.133	56	2	2010
Almutairi AF	2	2	0.286	8	2	2018
Alvarez-Seguí M	2	2	0.095	50	2	2004
Alvarez M	2	2	0.087	35	2	2002
Badiye A	2	3	0.222	41	3	2016
Bhuyan L	2	2	0.4	6	2	2020
Caldas IM	2	3	0.111	220	3	2007
Crispino F	2	2	0.154	27	2	2012
Daruka Prasad B	2	2	1	33	2	2023
Doroz R	2	2	0.286	26	2	2018
Fernandes R	2	2	0.286	11	2	2018
Fonseca GM	2	4	0.182	32	4	2014
Gupta S	2	4	0.143	21	5	2011
Houck M	2	2	0.154	27	2	2012
Kapoor N	2	3	0.222	41	3	2016
Kurniawan A	2	2	0.5	14	2	2021
Malleshappa J	2	2	1	33	2	2023
Mamatha GR	2	2	1	33	2	2023
Mcadam T	2	2	0.154	27	2	2012
Nagabhushana H	2	2	1	33	2	2023
Porwik P	2	2	0.286	26	2	2018
Radha Krushna BR	2	2	1	33	2	2023
Salam M	2	2	0.286	8	2	2018
Sandhya S	2	2	0.286	11	2	2018
Shamim T	2	2	0.105	34	2	2006
Wrobel K	2	2	0.286	26	2	2018
Al-Kheraif AA	1	1	0.25	42	1	2021
Abdel Aziz MH	1	1	0.111	16	1	2016
Abedi M	1	1	0.333	5	1	2022
Abirami MS	1	1	0.5	1	1	2023
Acharya AB	1	2	0.053	27	2	2006
Adamu LH	1	1	0.1	10	1	2015
Afoakwah C	1	1	0.333	5	1	2022
Afonso A	1	1	0.056	189	1	2007
Ahamed A	1	1	0.125	1	1	2017
Ahmad R	1	1	0.333	12	1	2022
Ahmed A	1	1	0.167	3	1	2019
Ahmed Mujib BR	1	1	0.125	8	1	2017
Al-Rawashdeh N	1	1	0.2	2	1	2020
Al Samahi HAM	1	1	0.167	3	1	2019
Alaparthi R	1	1	0.1	5	1	2015
Aleidan HN	1	1	0.143	6	1	2018
Alhabib AA	1	1	0.143	6	1	2018
Alkhaldi CKH	1	1	0.167	3	1	2019
Alkhtheri BA	1	1	0.143	6	1	2018
Almadhani A	1	1	0.167	3	1	2019
Almasry SM	1	1	0.091	8	1	2014
Alotaibi EA	1	1	0.143	6	1	2018

Journals

**Figure 4:**
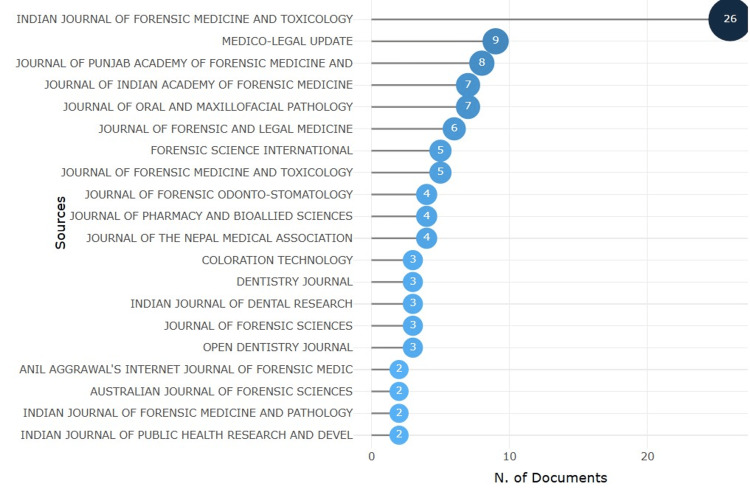
Journals with the highest number of publications

Figure 4 shows the most significant sources associated with lip print analysis. The Indian Journal of Forensic Medicine & Toxicology has the highest number of documents (26), followed by Medico-Legal Update with nine articles. The journal of the Punjab Academy of Forensic Medicine and Toxicology and the Journal of Indian Academy of Forensic Medicine each contributed eight and seven articles, respectively, emphasizing their strong interest in forensic medicine. The Journal of Oral and Maxillofacial Pathology published seven articles, while the Journal of Forensic and Legal Medicine contributed six as well. Forensic Science International and the Journal of Forensic Medicine and Toxicology each published five articles. This distribution highlights the variety of journals contributing to the specialized field of lip print research.

Top Cited Document

**Figure 5:**
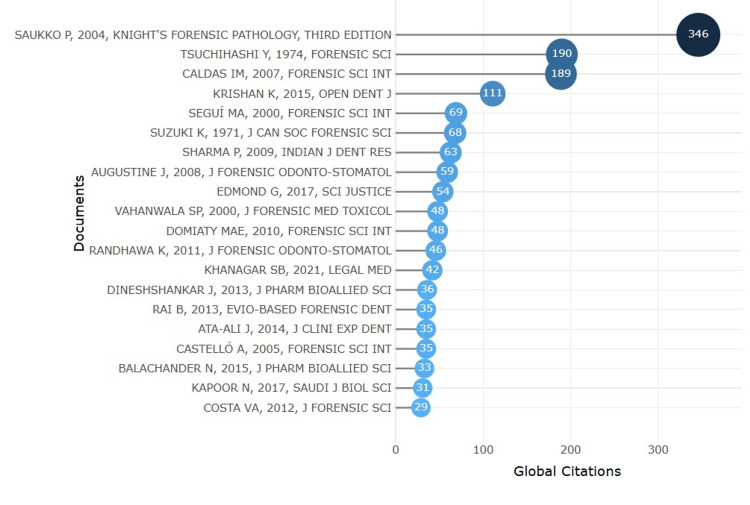
Documents with the highest number of citations

Figure 5 showcases the most globally cited documents in lip print and related forensic research. Topping the list is Saukko P, 2004, Knight's Forensic Pathology, Third Edition, with an impressive 346 citations, highlighting its foundational role in forensic science. Following this are Tsuchihashi Y, 1974, Forensic Sci Int with 190 citations and Caldas IM, 2007, Forensic Sci Int with 189 citations, both reflecting their significant contributions to the field. Other notable entries include Krishan K, 2015, Open Dent J (111 citations) and Seguí MA, 2000, Forensic Sci Int (69 citations). The chart also highlights various influential publications from journals like the Journal of Forensic Odonto-Stomatology, Indian J Dent Res, and Sci Justice. These citations underscore the importance of a few key works in advancing research and practice within the domain of dental age estimation.

Keywords

**Figure 6:**
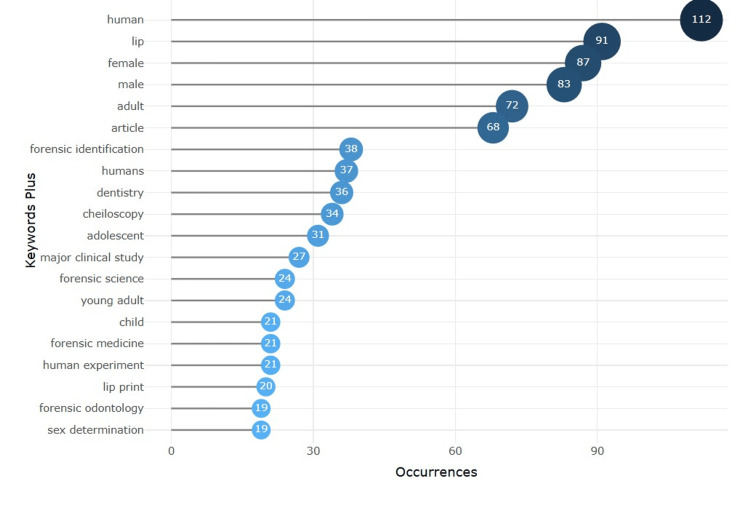
Highest occurrences of keywords

**Figure 7:**
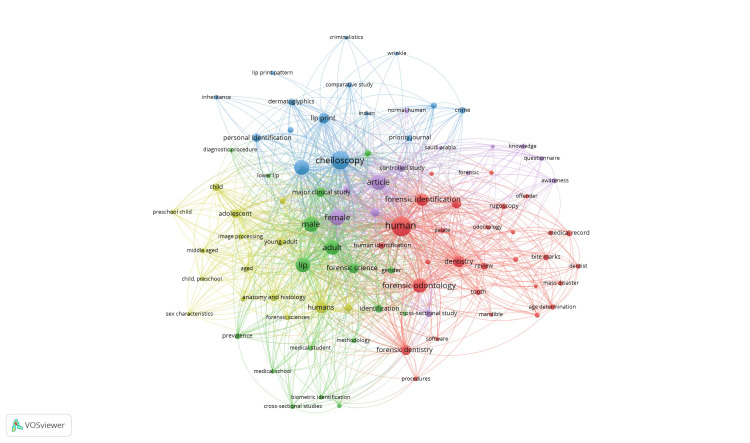
Co-occurrence of keywords

Figure 6 shows the keyword frequency analysis highlights the primary focus areas in forensic research, emphasizing human identification through specialized techniques. The most frequent terms include "human" (112 occurrences), "lip" (91), and demographic indicators such as "female" (87), "male" (83), and "adult" (72), reflecting a focus on population-specific studies. Notable forensic terms like "forensic identification" (38), "cheiloscopy" (34), and "forensic science" (24) underscore the relevance of lip prints and related methods. Additionally, keywords such as "adolescent", "child", and "sex determination" illustrate the application of these techniques across diverse age groups, reinforcing their clinical and forensic significance. Figure 7 shows the co-occurrences of keywords in which the highest is shown by the keyword “human”, which had 112 occurrences and 88 links with a total link strength of 1,286. This is followed by the word “male”, which had 96 occurrences and 87 links with a total link strength of 829.

Bibliographic Networking

**Figure 8:**
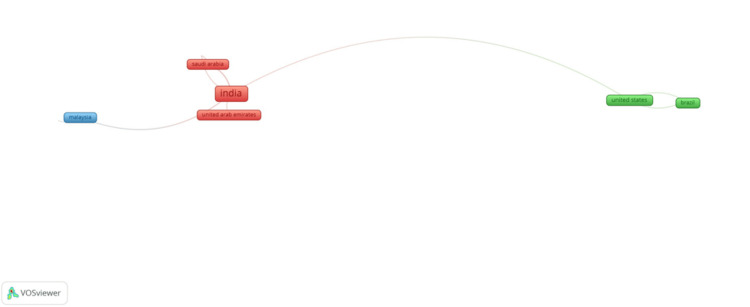
Bibliographic networking of co-authorship with countries

Co-authorship with countries: Figure 8 represents the networking map of co-authorship and countries. Out of all the authors involved, only 10 countries met the threshold. This map shows a total of nine clusters all over the world. The country that has the most number of co-authors in publications is India with 121 documents, five links, and eight total link strength. This is followed by United States having 11 documents, three links, and three total link strength.

**Figure 9:**
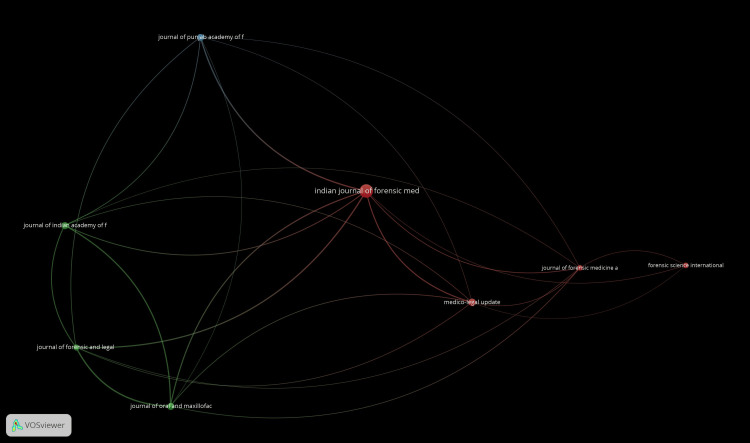
Bibliographic coupling of sources

Bibliographic coupling of sources: Figure 9 shows the networking map of bibliographic coupling of sources. Out of all the sources, only eight met the threshold. This map shows a total of eight clusters all over the world. The Indian Journal of Forensic Medicine and Toxicology shows 26 documents, seven links, and 89 total link strength. This is followed by Medico-Legal Update, which shows nine documents, seven links, and 35 total link strength.

**Figure 10:**
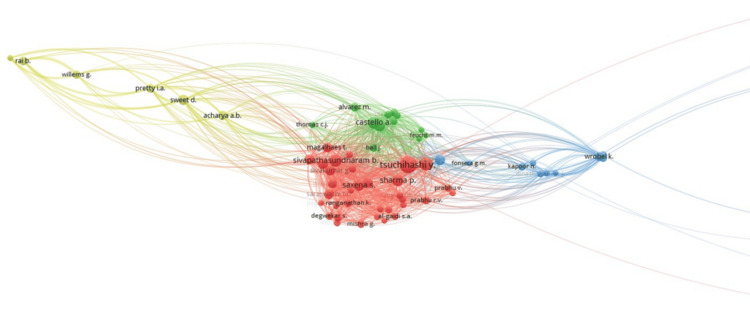
Bibliographic networking of co-citation of authors

Co-citation of authors: Figure 10 shows the co-citation of authors. Out of all the authors, 66 authors met the threshold with five clusters, 1,750 links, and 29,840 total link strength. Tsuchihasi Y showed 165 citations, seven links, and 89 total link strength. This is followed by Sivapathasundharam B, which shows 87 documents, 58 links, and 1,807 total link strength.

Discussion

This manuscript highlights the development, important authors, and research tendencies of lip prints and related forensic studies from 1971 to 2024. The bibliometric analysis indicates a constant increase in the number of publications with a growth rate of 3.08%, representing an increasing interest in this area worldwide. The remarkable increase in publications between the years 2004 and the year 2020 and the year 2022 has been apparently due to innovations in forensic identification techniques, including but not limited to increasing applications of cheiloscopy in legal and medical investigations [9,10].

India is the highest contributor with 133 publications, which reflects strong academic interest in forensic odontology and considerable research funding in forensic sciences in India [11]. In addition, contributions from countries such as Saudi Arabia and Chile, which have received high MCP ratios, underscore the role of international collaboration in developing this niche area of research [12]. The reasons for this may lie in the growth of educational systems in India in forensic odontology, which has witnessed a heightened awareness regarding its significance within criminal justice processes and disaster victim identification. Research funding allocated to forensic sciences by India represents a commitment at both government and institutional levels toward development within the discipline. The very high MCP ratios with Saudi Arabia and Chile reinforce the idea that international collaboration not only facilitates knowledge transfer but also showcases resource-sharing opportunities and access to diverse case studies, which are indeed vital for advancement as a global discipline.

The keyword analysis reveals the precedence of words such as "human", "lip", and "forensic identification", indicating that lip prints remain central in any form of human identification [13]. Keywords co-occurring most often with demographic factors are "male", "female", and "adult". These techniques are applied across population subsets for sex and age estimation, which is an essential part of forensic investigation [14]. The presence of the key term "cheiloscopy" with a high frequency reinforces that it is regarded as a reliable and non-invasive identification method [15].

Despite the increasing number of articles published, the decline in average citations per article in recent years indicates that research is being disseminated more broadly rather than being conducted on highly impactful studies. This trend means that although the discipline broadens, further efforts must be made to improve the quality and citation impact of the research [16].

Recognized institutions in the field, such as Saveetha University and Ajman University, have made significant contributions to demonstrate their commitment to forensic sciences. The narrower focus on forensic odontology at these institutions has led to their designation as centers of excellence in this very specialized area [10]. The Indian journals, such as the Indian Journal of Forensic Medicine and Toxicology, indicate the regional orientation of research activity on this subject [10]. Such a concentration of contributions from Saveetha University and Ajman University is due to their dedicated programs with an emphasis on incorporating forensic odontology into developed curricula and academic research projects. These establishments in faculty expertise, research facilities, and collaborations with government and the private sector presumably led to their being designated as centers of excellence. In addition to the above, having significant regional journals like the Indian Journal of Forensic Medicine and Toxicology also stresses the need for indigenous publishing in order to promote research that is region-centered as well as indigenize the forensic science applications demanded from various local requirements.

The networking analysis illustrates the diverse but developing collaborative panorama. India occupies a leading position in the international research network due to its massive output of papers and co-authorships. At the same time, the bibliographic coupling and co-citation networks are clustered, indicating interconnectedness in this area, especially in regions where established forensic institutions exist [17].

Notably, among the most cited documents are such fundamental works as Knight's Forensic Pathology and the early cheiloscopic studies by Tsuchihashi Y, which have significantly contributed to the scientific comprehension and practical utilization of lip print analysis [18]. These illustrations emphasize the importance of a solid groundwork that supports later developments.

This study has certain limitations that must be acknowledged. First, the analysis is reliant on bibliometric data, which may not capture the full spectrum of research activities, particularly those published in non-indexed journals or in gray literature. Second, the study focuses primarily on publication metrics, which might not reflect the actual impact or quality of the research. Additionally, language bias may exist as non-English publications are often underrepresented in bibliometric databases. Furthermore, while collaboration metrics were explored, the factors that motivate these collaborations were not deeply examined; thus, a particular institutional or regional dynamic cannot be understood.

Despite these limitations, the study offers several notable strengths. It provides an exhaustive bibliometric overview of forensic odontology research regarding global trends, main contributors, and new areas of collaboration. The inclusion of particular metrics like MCP ratios and institutional rankings makes the analysis more profound by giving an idea about the collaborative dynamics as well as academic excellence in this niche field. Besides, the study will foster less explored regions and institutions for a better overall picture of the global research endeavor in forensic sciences.

This study is relevant to a diverse group of stakeholders, including researchers, policy-makers, and educational institutions. It will pin down the key players and collaborative networks with an effective approach to enhancing international cooperation and building up capacities in forensic odontology. Findings will also provide justification for investing in targeted areas underrepresented to correct global disparities in research in forensic science. Furthermore, such a study may help researchers find influential journals and institutions that could publish their work and develop forensic odontology as an important interdisciplinary field.

## Conclusions

In conclusion, this bibliometric analysis emphasizes the dynamic and evolving character of forensic odontology research, highlighting India as an important contributor with notable contributions from institutions such as Saveetha University and Ajman University. The findings herein serve to underscore the importance of regional and international collaborations in advancing this specialized field. While both publication bias and the singular focus on bibliometric measurements have been called out as limitations, the broad insights of the study regarding publication trends, contributions by institutions, and collaborative efforts make it quite a handy resource for shaping the future of forensic odontology research worldwide.
